# Optimizing Social Network Support to Families Living With Parental Cancer: Research Protocol for the Cancer-PEPSONE Study

**DOI:** 10.2196/resprot.5055

**Published:** 2015-12-30

**Authors:** May Aasebø Hauken, Mette Senneseth, Atle Dyregrov, Kari Dyregrov

**Affiliations:** ^1^ Center for Crisis Psychology Bergen Norway; ^2^ University of Bergen Faculty of Psychology Bergen Norway; ^3^ Bergen University College Faculty of Health and Social Sciences Bergen Norway

**Keywords:** randomized controlled trial, parental cancer, children, social support, psycho-education, quality of life, mental health

## Abstract

**Background:**

Parental cancer can have a significant impact on a family's psychosocial functioning and quality of life, whereby the children’s situation is strongly related to parental coping and capacity. Such parents ask for more help in order to increase their care capacity, while the network is often insecure about how to help and thereby withdraw. They ask for guidance and training to be able to support cancer families. Based on this, the Cancer- Psycho-Educational Program for the SOcial NEtwork (PEPSONE) study was developed.

**Objective:**

To optimize social network support through a psycho-educational program for families living with parental cancer and their network members in order to increase parental capacity and thereby secure the children’s safety and quality of life.

**Methods:**

A randomized controlled trial (RCT) in which families (N=60) living with parental cancer will be randomized to either an intervention group or a control group. The intervention will last for 3 hours and includes (1) introduction, (2) psycho-education (living with cancer in the family and the importance of social network support), and (3) discussion (this family’s need for social support). Primary outcomes are social support, mental health, and quality of life, and secondary outcomes are resilience and parental capacity. Data will be collected by a set of questionnaires distributed to healthy parents (N=60) living with a partner with cancer, one child in the family between 8-18 years of age (N=60), and network members (N=210) of the intervention families at inclusion, and after 3 and 6 months. Comparing differences between the intervention group (n=30) and the control group (n=30), the power analysis shows that *P*<.05 and a statistical power = .80 would detect effect sizes of clinical interest.

**Results:**

This paper presents the Cancer-PEPSON study’s protocol to provide a broader understanding of the background and content of the program. The study is ongoing until August 2016 and the first results are anticipated to be finished by November 2015.

**Conclusions:**

To our knowledge, this will be the first RCT study to optimize social network support through a psycho-educational program for families living with parental cancer and their network members, as well as provide an evidence basis for social network support. The results may provide important knowledge that is useful for clinical practice and further research. The trial is reported according to the CONSORT checklist.

**ClinicalTrial:**

International Standard Randomized Controlled Trial Number (ISRCTN): 15982171; http://www.controlled-trials.com/ISRCTN15982171/15982171 (Archived by WebCite at http://www.webcitation.org/6cg9zunS0)

## Introduction

Annually, approximately 30,000 Norwegians are diagnosed with cancer. Even if the majority of these are older individuals, more than 3500 children under <18 years of age experience a parent getting cancer. Thus, about 18,000 Norwegian families live with parental cancer [1,2]. Internationally, roughly 14-18% of cancer patients have dependent children, indicating a large population of families for whom cancer poses special challenges [3-5].

Social support is important for human health and quality of life, including emotional, practical and economic help, and information provided to the individual by significant others, such as distant family members, friends, and co-workers, etc [6]. Even if social support represents an essential resource for families living with parental cancer, these parents report a need for more social support and help in order to uphold their parental capacity and to continue a “normal” everyday life [7-9]. Social network members want to support and help, but request support assistance from professionals to provide them with knowledge and various strategies to facilitate better and more prolonged support [7,10]. Based on this, we developed the Cancer- Psycho-Educational Program for the SOcial NEtwork (PEPSONE) study. Cancer-PEPSONE is a randomized controlled trial (RCT) study aimed at optimizing social network support through a psycho-educational program for families and their network members, in order to uphold parental capacity and children’s quality of life. This paper presents the study’s protocol to provide a broader understanding of the background and content of the study.

### Previous Research

In a demanding balance between caring for children, work, and domestic tasks, cancer illness and cancer treatment represent a significant burden and long-lasting strain for the entire family [4,11,12]. These consequences are, however, different for individual family members.

A cancer diagnosis and treatment usually results in multiple consequences for the sick parent, including physical and psychosocial side effects, such as nausea, pain, and fatigue as well as anxiety, depression, and traumatic stress reactions [13,14]. Sick parents are, therefore, frequently challenged in fulfilling their roles in everyday life in relation to work, practical tasks at home, and caring for their children and social life [7,15]. Partners of cancer patients also experience stress reactions, anxiety, depression, and impaired quality of life [16-18]. They frequently report a considerable increase in the strain related to child care, in performing domestic tasks, and in supporting their sick partner as well as being the family’s breadwinner. Healthy partners often fulfill double roles and feel distressed, insecure, and lonely [7,11,19]. Together, these challenging strains on both parents may negatively influence their parental capacity and quality of life [12,20,21].

How children are affected by parental cancer depend on the child’s age, experiences and maturity, and the cancer severity, but are especially related to parental coping and the family’s function in everyday life [4,11,22]. Reduced parental coping negatively affects the children’s behavior and their emotional, physical, and school life [23,24]. Young children are mostly affected by concrete changes in daily routines such as frequent hospitalizations and changes in parental behaviors (eg, sadness, fatigue, and impatience) [25]. Older children and adolescents also feel empathy, and they are worried about losing their parent and think about how the cancer will influence their own futures [23,26]. These children are at risk of several physical and psychosocial symptoms such as decreased energy levels, headaches, stomach pain, sleep deprivation, concentration problems, depression, anticipatory grief reactions, and reduced quality of life [19,24]. Additionally, these children report impairments in the family’s social life and increased involvement in domestic tasks such as looking after minor siblings [23]. Research indicates that they try to reduce the strain on parents by asking for less help or not bringing friends home, as well as internalizing their own problems and concerns [23,24]. Thus, children living with parental cancer ask for predictability and stability in everyday life, and call for a balance between talking about their current situation and a “space” in which they can talk about things other than cancer, hang out with friends, and participate in leisure activities [7,27].

Several studies have emphasized the importance of social support for physical and mental health, for quality of life, as well as for coping with and recovering from cancer [28-31]. Nonetheless, cancer patients still experience disruptions in their social lives as well as unhelpful help or a lack of social support, especially over time [8,32]. Cancer patients’ main concern regards caring for their children, and in particular they want to protect their children and maintain an ordinary everyday life [20,33,34]. A number of these parents express that they are dependent on help from their social network to sustain their regular everyday life, and call for more and prolonged support to uphold their own parental capacity and avoid “hitting the wall” [7,35]. These families often find it difficult to ask for the help and support they actually need and they often experience that help and support drop shortly after the diagnosis and their network withdraws [7-9].

Previous research has found that network members surrounding families in crisis are generally positive in providing support but that they are often insecure about how to help. They report being afraid to say or do the “wrong things”, of intruding on the family, or they may assume that their contributions are unimportant or that the family copes adequately on its own [7,8,36]. Network members claim that by acknowledging support efforts and providing various strategies, professionals can facilitate better and more prolonged network support [36].

Most studies of social support related to cancer are descriptive, documenting the importance of social support for physical and psychological health as well as for quality of life [32]. Intervention studies on enhancing social support lag far behind, and are mostly directed to cancer patients, especially breast cancer, focusing on different kinds of support groups [37,38]. Intervention studies related to parental cancer and dependent children are lacking, as are intervention efforts aimed at enhancing social support for children and families members [39]. However, Hogan’s review [38] provides support for the overall usefulness of different social support interventions.

### Theoretical Framework

Social support is thought to affect mental and physical health positively through its influence on emotions, cognitions, and behaviors [6,38,40]. Nevertheless, the association between social support, well-being, and health is complex and therefore difficult to conceptualize. House and Kahn’s definition of social support makes a conceptual distinction between different types of social support, including emotional, economic and practical help, and the provision of information [41]. Social network support encompasses various kinds of support given to individuals and families by other family members, friends, colleagues, neighbors, and others [6,11]. Over the last decades, much health research has distinguished between “perceived” and “received” social support [6,42]. “Perceived support” refers to the perception by those who are in need of support that such support would be available if needed (ie, qualitative), whereas “received support” refers to the actual support resources received by those in need (ie, quantitative). Two major models have been proposed to explain the link between social support and well-being: the main or direct effect model and the buffering effect model [6,43]. The direct effect model suggests that social support is directly associated with well-being, while the stress buffering model describes how social support can protect individual well-being from the negative impact of stress. Perceived and received support have exhibited different effects on well-being and health, with perceived support demonstrating the most influential effect [6]. Furthermore, social support has been seen as transactional, which means that other factors (eg, personality characteristics, contextual, and interpersonal processes) influence the impact of the support. Nevertheless, exactly what the mechanisms are which provide for the effects of social support remains to be discussed and the causal links are still unclear [6,42].

**Figure 1:**
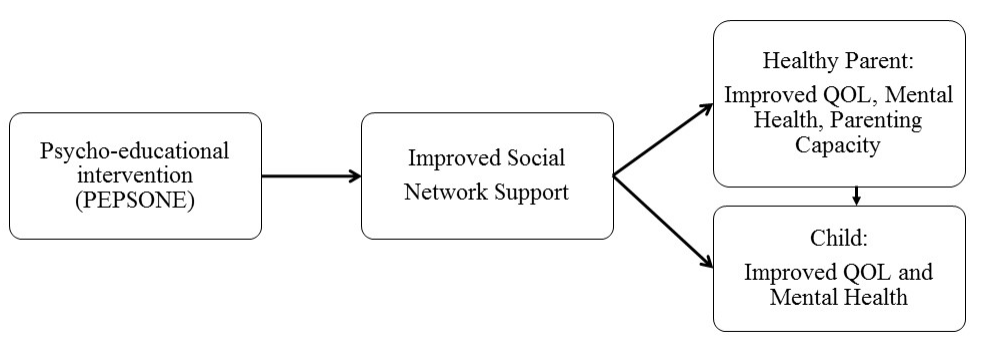
Research model.

Cohen [42] argues that strengthening and increasing the availability of support in social networks, and reducing negative interactions within one’s network are essential for human health. Psycho-education can be a viable strategy for achieving this. Psycho-education is defined as professionally delivered illness-specific information and tools for managing challenges in everyday life [44]. It builds on a holistic and competence-based approach, focusing on health promotion, collaboration, and empowerment where the development of open communication, competence, knowledge, and skills are crucial elements facilitating behavioral change [44]. Psycho-education looks to support the individual's understanding of a challenging situation and help gain access to resources, develop awareness of issues, foster a sense of control, and educate about coping skills for both families and their networks. These factors focus on improving cognitive awareness and coping skills. However, psycho-education also looks to promote insights that address affective worries and concerns [44]. This study focuses on investigating whether the psycho-education of social networks’ members is an appropriate method to achieve beneficial effects with respect to increasing social support and thereby parental quality of life, mental health and parental capacity, and thus the children’s well-being. Based on the theoretical framework we have developed a conceptual model of the study (Figure 1).

As shown in Figure 1, it is hypothesized that the Cancer-PEPSONE program will enhance the family’s social network support. This enhanced support will have direct effects on healthy parents’ quality of life, mental health, and parental capacity, as well as direct and indirect effects on children’s quality of life and mental health. The study upholds the child’s perspective and complies with the Norwegian Act for Health Personnel [45], putting children’s well-being on the agenda when living with parental illness. It also complies with the International Convention on the Rights of the Child (UN), emphasizing the rights of children to care, protection, rehabilitation, and assistance in various situations that can negatively affect their life situation.

### Objectives and Hypotheses

The purpose of the Cancer-PEPSONE project is to expand the knowledge base and build competence in networks to help children living with parental cancer. The overall aim of the study is to optimize social support from the social network through a psycho-educational intervention. Based on the project’s aims and research model, we hypothesize that (1) a psycho-educational program will improve the provisions of social support to the affected family, (2) parental psychosocial health and quality of life will increase through social network support, and (3) the children’s psychosocial health and quality of life will improve because of more and better social support and increased parental capacity, mental health, and quality of life.

## Methods

### Trial Design

**Figure 2:**
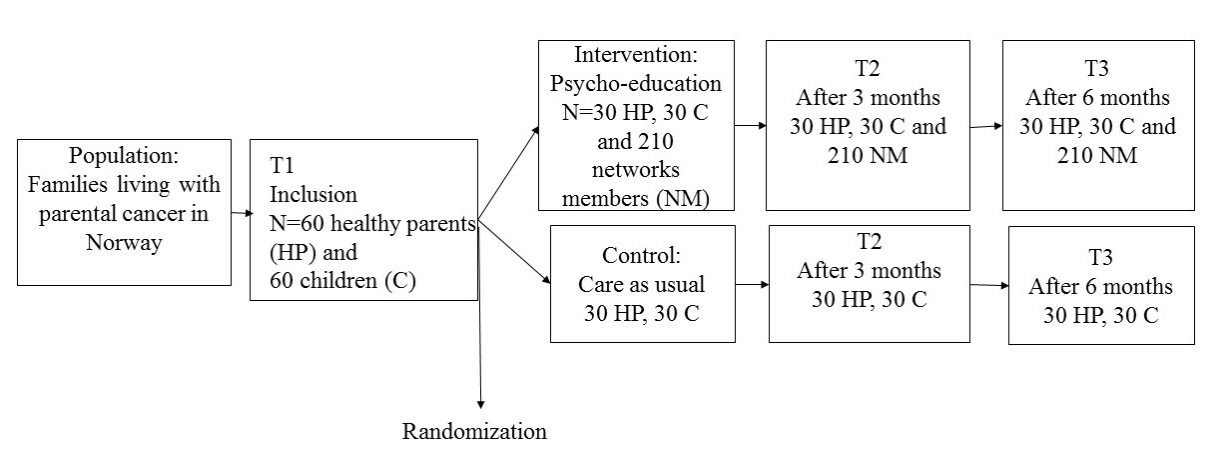
Flow diagram of the The Cancer PEPSONE study.

Based on the study’s hypothesis and research questions, the Cancer-PEPSONE study will be conducted as a single center, randomized controlled trial (RCT) [46], including an intervention and a control group. This design is best suited to test the effectiveness of various types of intervention in clinical trials. The Cancer-PEPSONE study design is outlined in Figure 2.

After receiving written and oral information from one of the two first authors, families fulfilling the inclusion criteria are included in the study. The participants receive a form for informed consent and the first set of questionnaires for the healthy parent and one child in the family (T1) by mail. The consent form and the questionnaires are returned to the researchers in pre-stamped envelopes. Then, every other family is randomized to either the treatment group or the control group by one of the researchers based on the order in which the informed consent and questionnaires are returned. The type of intervention makes it impossible to blind the trial participants or the psychologist. The families in the intervention group and their network members receive the intervention, while the control group receives no intervention (care as usual). The control group is offered the intervention after finishing the study participation, after approximately 6 months, and all participating families get a DVD of the program after finishing T3 after 6 months.

### Intervention

The intervention is a psycho-educational program for the family and its network members. The authors developed the program based on clinical experience, previous research, and the theoretical framework previously outlined. It was pilot-tested by two of the authors and thereafter, all the authors discussed and modified the program to its final structure.

The parents in the intervention families decide who in the family and which network members would participate in the program. The program is conducted in the families’ homes, or else where they choose, by one of three clinical psychologists, all of whom are experienced in working with families and children in crisis. It lasts for approximately 3 hours. None of the researchers are involved in the intervention that contains the elements described in Textbox 1.

**Elements included in the intervention.**

- Welcome and introduction (10-15 minutes): The psychologist leading the meeting introduces herself and presents the content of the program before the participants introduce themselves. The goals of the program are (1) to emphasize the importance of network support; (2) secure social network support for cancer families over time; (3) share knowledge to make each other wiser.
- Psycho-education (approximately 1 hour): In this part, the psychologist has a teaching session focusing on the following areas: (1) the consequences of living with a cancer diagnosis in the family for the sick and the healthy parent, and with a special focus on the children; (2) general reactions and the needs of both the children and the adults; (3) common useful coping strategies for crisis; (4) the importance of social network support, focusing on what is social network support for families in crisis, and what do we know about helpful network support; (5) the importance of “openness” and communication between the family and the network; (6) what can social networks do, and different types of social support (eg, emotional, practical, information, and economical support); (7) how to sustain network support over time: the importance of distribution, and the coordination of support.
- Discussion (approximately 1.5 hours): Based on the teaching session, the goal of the discussion is to enhance the family and its network members’ understanding of the value of open communication about the family’s need for social support and the network members’ ability and willingness to give such support. The psychologist facilitates the discussion based on the experiences from previous support giving/receiving processes between the family and the network, the family’s current needs and what is the network able and willing to do as well as coordination of the network support.
- Summing up and closing (10 minutes): The psychologist sums up the main points from the teaching session and the discussion.

A detailed procedure for the intervention is developed and reviewed by the intervention psychologists together with the authors securing that the intervention is performed in the same manner for all families. After the meeting, the psychologists fill out a form with information about how the intervention went according to the protocol, who attended the meeting (roles/relations), the themes discussed, and a short field note to record any observations about the context and impressions arising from the meeting. All participants in the intervention also fill out an evaluation form on how they experienced the psycho-education.

### Eligibility Criteria

The study contains 3 samples. Sample 1 and 2 (n=60) consist of 30 families in the intervention group and 30 in the control group. These samples include the healthy parent and one child from each family. The inclusion criteria for these families are (1) a healthy parent having a partner or spouse diagnosed with cancer within the last five years and treated for cancer and (2) one child in every family, aged 8-18 years old, living with a parent who has cancer. With multiple children in the family, the oldest child who is willing to participate will be recruited. The parents in the intervention group ask the number of adult network members (mean 7, limited to 15 network members, N=210) if they want to participate in the intervention. This group of adult network members makes up Sample 3. The inclusion criteria for these network members are (1) extended family members, friends, neighbors, and work colleagues of the parents, (2) ≥18 years, and (3) living nearby the family.

The exclusion criteria for the study are (1) healthy parent not living with the ill parent or the ill parent has died, or having a serious disease, (2) children <8 years old, not living with ill parents, serious disease themselves, and (3) network members living >2 driving hours from the family.

#### Recruitment

Participants are recruited nationwide using a wide-ranging recruiting strategy including information acquired through hospitals and primary healthcare, brochures, and different websites. Families are also recruited through the Norwegian Cancer Society, the Montebello Cancer Center, child responsible healthcare professionals in hospitals, cancer coordinators in primary healthcare, and resource nurses in cancer care and palliation.

#### Outcome Measurements and Data Collection

The literature recommends using a range of outcome measures in evaluating complex interventions, as a single outcome may not capture the results or unintended consequences of the study [47]. Therefore, different self-reported questionnaires are included for all participants, where social support, mental health, and quality of life are primary outcomes, while resilience and parent capacity are secondary outcomes.

##### Outcome Measurements

The questionnaire for the healthy parents includes the information shown in Textbox 2. The questionnaire to the children includes the information shown in Textbox 3. The questionnaire set to the network members includes the information shown in Textbox 4.

**Information included in the questionnaire for the healthy parents.**

- Demographic data about the healthy parent: age, gender, education, social status, children, employment status, and income.
- Demographic and medical data about the sick parent: age, gender, type and degree (metastasis) of cancer, months since diagnosis, type of treatment, and months of treatment and current treatment status.
- The Crisis Support Scale (CSS) [48] and the Assistance Questionnaire-Receivers of support (AQR) [49] measure social support. CSS is a short scale for measuring social support after a crisis has occurred, consisting of 7 questions with a rating scale from 1 (never) to 7 (always). All items are summed for a total mean score, where a higher total score indicates more received support. The scale appears to be very robust and to have satisfactory psychometric properties [48]. AQR measures adults’ experiences and need for 9 different types of social support related to the situation caused by the cancer. The instrument was developed by Dyregrov et al [49] and is applied on comparable populations both nationally and internationally.
- The Quality of Life Scale (QOLS-N) is used to measure the healthy parent’s quality of life [50]: This instrument measures an individual’s overall satisfaction with life based on different life domains. It contains 16 items scored on a 7-point Likert scale ranging from “very satisfied” to “very dissatisfied”. The items are calculated into the 6 subscales to assess satisfaction with life domains. The dimensions are scored by summing the scores for each item in the subscale. Possible total scores range from 16-112, where a lower score indicates worse quality of life demonstrated satisfactory psychometric properties in several studies [50,51]. The 6 subscales are as follows: (1) physical and material well-being; (2) personal development; (3) relationships with others; (4) participation in social activities; (5) participation in community and civic activities; and (6) recreation.
- The General Health Questionnaire (GHQ-12) is used to assess current mental health and psychological distress, reflecting the inability of normal functioning in regard to distressing experiences [52]. The questionnaire consists of 12 items on a Likert scale ranging from 0 to 3, where a higher sum-score indicates more symptoms of psychological distress and worse mental health. The GHQ-12 is widely used as a reliable screening instrument for psychological distress and minor psychiatric morbidity outside a clinical setting, showing high psychometric properties in various populations [52].
- The Self- Efficacy Parent Task - Short Form (SEPTI-SF) is used to measure 2 dimensions of the parent’s self-efficacy, discipline and achievement, showing satisfactory psychometric properties [53]. These dimensions consist of 11 quotes with 6 alternative answers from “highly disagree” (1) to “highly agree” (6). The dimensions are scored by summing the scores for each item in the subscale.
- The Dispositional Resilience Scale-Revised (DRS-15-R/Hardiness) is used to assess the parents’ hardiness in meeting challenging life events and situations [54]. The questionnaire consists of 15 quotes with 4 alternative answers scored on a Likert scale ranging from “highly disagree” (0) to “highly agree” (3). The items are summed into the 3 dimensions, commitment, challenge, and control, where a higher score indicate higher hardiness. The instrument is used internationally as well as in Norway, and has demonstrated validity and reliability within a wide range of studies as well as sensitivity to change [54].

**Information included in the questionnaire to the children.**

- Demographic data: age, gender, siblings, and grade in school
- The Revised Child Manifest Anxiety Scale (RCMAS) is used to measure anxiety reactions in children [55]. The RCMAS consists of 28 anxiety items and 9 lie (social desirability) yes-or-no items. Sum scores are provided for total anxiety and the 4 sub-scales: worry/oversensitivity, physiological anxiety, social concerns/concentration and a lie scale. A higher score indicates higher levels of anxiety or lie. RCMAS has demonstrated adequate psychometric properties [55].
- Kinder Lebensqualität (KINDL) is used to assess the quality of life of children [56]. KINDL consists of 30 quotes with 4 alternative answers ranging from “never” to “always”. The 6 subscales (physical health, emotional well-being, self-esteem, family, friends, and school) are calculated, where higher scores indicate higher QOL. The questionnaire has showed psychometrically acceptable values [56]. KINDL has 3 versions related to age, but since these cover the exact same questions with somewhat different wording, we decided to use Kid-KINDL to cover the entire age-span.

**Information included in the questionnaire to the network.**

Questionnaire information

- Demographic data: age, gender, education, social status, work affiliation, and relation to the family/sick parent.
- Assistance Questionnaire: Providers of support (AQP) represents the opposite version of AQR as it measures the social support given.
- GHQ-12, QOLS-N, Hardiness and CSS, as described for the healthy parents.

##### Data Collection

Healthy parents and the children fill out the entire dataset at inclusion (T1), after 3 months (approximately one months after the intervention for the intervention group), and after 6 months (Figure 2). One of the two first authors provides the families with questionnaires by post and they return them to the researchers in pre-stamped envelopes. The social network members in the intervention group fill out the T1 questionnaires prior to the meeting starting. The network members in the intervention group fill out the same questionnaires after 3 (T2) and 6 months (T3), distributed via the Internet and the website SurveyMonkey.

##### Power Calculations and Statistical Analyses

When comparing differences between the intervention group (n=30) and the control group (n=30), the power analysis showed that with *P*<.05 and a statistical power = .80, one would be able to detect effect sizes of *t* tests of about 0.65 of one standard deviation or higher [46]. This means an ability of detecting effects sizes of medium size or higher according to Cohen’s criteria [46]. This effect level was judged to be of clinical interest, and was within the study’s recruitment frame and economy.

All the data will be coded, verified, and entered into IBM SPSS Statistics for Windows Version 22.0. Normality will be assessed through examinations of skewness and kurtosis for all variables. A two-tailed *P* value of <.05 is considered to be statistically significant [46,57]. Descriptive statistics (mean/median or percentages, SD, and ranges) and correlation analyses (Pearson correlation) will be used to describe the data and to explore relationships among them [57]. The outcome variables will provide sum-scores on the interval level. Multiple regression analysis will be used to explore directional relationships among variables and testing the research model. For the estimation of the effect and the relationships of the variables relevant to the intervention, different methods such as *t* tests and structural equation (SEM) analyses will be used [57]. The missing data problem will be analyzed according to the questionnaires’ manual. Statistics will be reported in line with the SAMPL guidelines stated by Lang and Altman [58].

#### Ethical Implications and Risk

The Regional Committee of Research and Ethics in Western (REK West) Norway and the Norwegian Social Science Data Services (NSD) approved the research protocol October 9, 2013 (reference number: 2013/1491/REK vest).

The study will be conducted in compliance with the Declaration of Helsinki [59] and the requirements for data processing outlined in the NSD [60]. Only the two first authors have access to the data files. Procedures for handling data and data security in the study are developed. Study participation is based on written and oral information and written consent [59]. For children <12 years of age, the parents must consent on behalf of the children, whereas for the 12-18 year old children the consent is given from both parts. Study participation is voluntary and participants can withdraw at any time without the provision of reasons or any negative consequences. Serious risks or undesired effects of the intervention or the assessment by questionnaires are not described in the literature and no specific risks related to this study are anticipated. All the professionals participating in the study have extensive experience as researchers or clinicians in the field of working with children, serious illness, crisis, grief, and trauma. This competence will secure ethical and safe conditions for the participants. A referral process for further assistance or treatment for participating families with special needs will be ensured by the psychologist. Any changes in the study protocol will be applied for to REK West and NSD.

#### Project Organization, Funding, and Timeframe

The Cancer-PEPSONE study is a one-center study conducted in Norway. The study is connected to an interdisciplinary research group and an international advisory board to provide input and secure the quality of the study, independent from the sponsors. The study is fully founded by the Research Council of Norway (4.7 million NOK) and by the Norwegian Directorate of Health (1.3 million NOK). The funders have no role in conducting the study, but reports on progress according to the protocol as well as economy are sent annually.

The study’s timeframe is 3 years. It was initiated in August 2013 and will be completed by August 2016. A schematic detailed timeline of the study is outlined in Table 1.

**Table 1:** Timeline for the Cancer PEPSONE study.

	Study period						
Time point	Autumn 2013	Spring 2014	Autumn 2014	Spring 2015	Autumn 2015	Spring 2016	Autumn 2016
Founding and ethical approval	x						
Advisory board	x	x	x	x	x	x	x
Research group meetings		x	x	x	x	x	x
Recruitment	x	x	x	x			
Enrollment		x	x	x			
Data collection T1		x	x	x			
Data collection T2		x	x	x	x		
Data collection T3		x	x	x	x	x	
Analyzing data					x	x	x
Publishing results						x	x
Study close-out							x

### Results

The Cancer PEPSONE study is ongoing, where enrollment of families began in January 2014. As of October 2015, 45 families are enrolled in the study. Of those families, 19 are randomized to the intervention group, with 15 families receiving the intervention together with approximately 120 network members, while 4 families are waiting for the intervention. As well, 20 families are enrolled in the control group and 6 families are not yet randomized. The first results from the study are expected in October 2015.

### Discussion

#### Principal Findings

The objective of this study is to optimize social network support through a psycho-educational program for families living with parental cancer and their network members in order to increase parental capacity and thereby secure the children’s safety and quality of life. Until now, the research regarding parental cancer has mainly focused on describing the parents’ challenges regarding their risk of impairment of psychosocial health, quality of life, and parental capacity [4,12]. In addition, they have described their positive and negative experiences with social network support [7,8]. Descriptive research documents that children living with parental cancer are especially exposed and vulnerable, and that their physical, psychosocial, and behavioral impairments largely depend upon their parents’ coping abilities [23,24]. In previous research, it is documented that it is a match between the bereaved and social networks’ accounts of the challenges involved in the support giving and receiving processes [10,36]. However, we lack intervention research to document the effect of systematic programs in order to optimize social support to make families living with parental cancer cope better with their situation. By including a family-, child-, and social network-perspective, the Cancer-PEPSONE study covers an important new area of research both nationally and internationally. Therefore, several important issues underpinning this study are worth highlighting.

The descriptive research has mainly focused on the negative consequences of parental cancer for both the sick and healthy parent as well as for their children. Consequently, these families’ experiences are at risk of being pathologized and assessed as being in need of individual professional treatment, instead of assistance in coping and building on their present resources [39]. Acknowledging that the lives for both the parents and the children involve more than just coping with the stress related to cancer, the Cancer-PEPSONE study builds on the inherent resources of family members and their networks, including a requested health promotion perspective into the research [39].

A strength of the Cancer-PEPSONE study is that it originates from the experiences brought forward in clinical practice and through our prior research. It builds upon the cancer families’ own outspoken need for more and long-lasting social support, as well as the networks calls for more knowledge and training from professionals as to how to provide good support and help over time [7,10]. The intervention is pilot-tested, securing its usefulness in clinical practice and meeting both the families’ and the networks’ needs.

Some may question the use of scarce healthcare resources to educate cancer families’ social networks. Professionals and researchers have gradually acknowledged that cancer patients live in a social context along with the need to provide psychosocial assistance during cancer more directly and to a greater extent than before [9]. Following legislation, Norwegian healthcare professionals have a statutory responsibility to ensure that the children of seriously ill patients receive information and follow-up during the entire illness trajectory [45]. This implies an obligation to secure psychosocial help for children, either directly or, most preferably, indirectly through parents by increasing their parental care capacity. This perspective is further elaborated in the Norwegian Cancer Strategy 2013-2017 [61], emphasizing a focus on help and support for both children and parents to be able to cope with their situation. These obligations highlight that health service must ensure that families receive assistance with practical, financial, and emotional issues as needed, as well as the municipality’s responsibility for coordinating this assistance.

Several families live with cancer over years that often involve irregular changes in the disease trajectory. Adjustment to cancer, therefore, involves a process rather than a singular event. A model that demands extensive professional follow-up over time would be very resource demanding. In contrast, by optimizing the “normal” and available source of social support, the use of formal resources may be limited. As such, limited use of professionals’ time spent in educating the families’ network members and promoting communication between the family and their social network seems preferable. According to our hypothesis, the psycho-education of the network members will lead to increased confidence in their interactions with friends and families in crisis. Hopefully, this will secure more long-lasting support and prevent “burn-out” in the networks. Most importantly, with more support, the parents may gain more capacity for caring for their children, and thereby prevent the negative consequences of living with parental cancer. Additionally, the improved and increased availability of social support will save society in financial expense.

From a resource perspective, it may also be questioned whether the intervention should be directed towards individual families and their networks, or else to several families and their networks at the same time. We consider this as a crucial element because families living with parental cancer are not a homogeneous group. Each family member faces special challenges and has different needs. Available network resources will also be heterogeneous. In line with this, the Medical Research Council [47] has stated the importance of tailoring complex intervention to local circumstances rather than being completely standardized. By meeting one family at a time, the psychologist can tailor the psycho-education to each family and its network, making sure that their perspective and context is at the basis of the information and conversation. By conducting the intervention in the family’s home, the importance of the family’s perspective and context is underpinned.

The intervention is four-fold, with an introduction, psycho-education, discussion, and closure. The introduction is important to set the content and the focus of the meeting, and for the participants to briefly get to know the psychologist and her competence. This allows for building trust and safety within the meeting. One goal of the psycho-education is to provide both the family and its network with knowledge of the specific challenges that adults and children face when living with parental cancer. Even if the family lives in such circumstances, this element can normalize their reactions, being revealing of the other family members’ challenges as well drawing focus on what is of most importance for the children. Many families find it difficult to express their own challenges and needs to their networks, and the psycho-education may therefore serve as recognition of their needs in preparing the ground for an open discussion later on. Another main goal of the psycho-education is the focus on the importance of social network support. Again, by providing knowledge to both the family and the network about the complex processes of social support, this may justify the family’s needs and give the network some helpful tools to use in the supportive interactions.

Most research has focused on the positive and helpful aspects of social support [6,30]. However, social support can also be experienced as negative or unhelpful, which is an issue that is important to focus on in both the psycho-education and in the discussion part of the intervention. Such negative social support can, for example, be non-helpful advices or advices that the family has not asked for, trivializing of their problems, or that the family feels overrun by help that they do not want or need [37]. In the discussion part, the psychologist builds upon the content of the psycho-education and tailors the content to the actual family and its network, discussing the most relevant aspects based on their situation. A clear goal is to facilitate direct, open and concrete communication between the family and its network members.

A possible limitation of the Cancer-PEPSONE study is that the intervention is short with no follow-up meetings, and therefore that it may be seen as naïve, with little potential to make any changes that last over time. Although the participants in the pilot study would have preferred 1 or 2 repeated meetings, the network members stated that the initial meeting had a good effect in sensitizing them to increased social support. In addition, resource allocation is an important argument. Thus, instead of organizing a follow-up meeting, the psychologists advise and trust the family and its network to organize the network to support themselves. An alternative might be that a local cancer nurse participates in the intervention and then follows up the family thereafter. It may be argued that it is not ethical if no follow-up is done for these vulnerable families. However, they are already enrolled in the public healthcare system, which have legislated obligation regarding follow-up [45]. If the researchers or the psychologists performing the intervention detect special needs of participating families, they will refer them to the public healthcare system. Another limitation of the study might be that we acquire very little knowledge about the qualitative and the procedural aspects of the intervention. However, by including a short evaluation of the program from both the psychologist, the family and the networks’ members, including open questions, we acquire more knowledge regarding these aspects. The study is founded as a RTC study. In addition, we will try to fund a qualitative arm of this study, exploring the participants’ experiences of both the intervention and its consequences.

The results from this study will be published in at least 6 papers in international peer-reviewed scientific journals, and 3 of these will be included in a PhD thesis. Furthermore, the findings will be presented in chronicles in national newspapers, at conferences, and seminars, both internationally and in Norway. Importantly, if the study results prove successful, we will develop guidelines for the Cancer-PEPSONE to be recommended for the local healthcare system and conducted by public nurses or cancer nurses, for example. The Cancer-PEPSONE program also has the potential to being adapted and studied related to other physical and mental conditions.

#### Conclusion

There is currently a lack of intervention studies related to parental cancer and children as well as intervention programs enhancing social support between families and social network members. Therefore, the overall aim of the Cancer-PEPSONE study is to optimize social network support through a psycho-educational program for the family and its network members in order to increase the parental capacity and thereby improve the children’s quality of life. It is anticipated that the results from this study will support the hypotheses and provide new knowledge about families living with parental cancer. The Cancer-PEPSONE study is innovative given the scope, including a family, a child, and a network perspective, and the intervention and the diversity of measures utilized within this longitudinal RTC design. We hope it will add to the growing body of research on children living with cancer in the family. We anticipate that, based on this study, we will be able to develop a guideline for the Cancer-PEPSONE program. By educating local healthcare professionals or volunteers in using such a guideline, the program is inexpensive and has the potential to be used in other parts of the world as well. By improving network support for these families, less help may also be needed from health professionals.

## Abbreviations

CSS: Crisis Support Scale
GHQ-12: General Health Questionnaire
NSD: Norwegian Social Science Data Services
PEPSONE: Psycho-Educational Program for the SOcial NEtwork
QOLS-N: Quality of Life Scale
RCT: randomized controlled trial
REK West: Regional Committee of Research and Ethics in Western Norway
